# Effect of Boron-Doped Diamond Interlayer on Cutting Performance of Diamond Coated Micro Drills for Graphite Machining

**DOI:** 10.3390/ma6083128

**Published:** 2013-07-25

**Authors:** Xuelin Lei, Liang Wang, Bin Shen, Fanghong Sun, Zhiming Zhang

**Affiliations:** School of Mechanical Engineering, Shanghai Jiao Tong University, Shanghai 200240, China; E-Mails: superlxl@sjtu.edu.cn (X.L.); liangwang@sjtu.edu.cn (L.W.); binshen@sjtu.edu.cn (B.S.); zhangzhiming1942@126.com (Z.Z.)

**Keywords:** diamond film, graphite machining, micro drill, interlayer, boron doping, cutting performance

## Abstract

Thin boron doped diamond (BDD) film is deposited from trimethyl borate/acetone/hydrogen mixture on Co-cemented tungsten carbide (WC-Co) micro drills by using the hot filament chemical vapor deposition (HFCVD) technique. The boron peak on Raman spectrum confirms the boron incorporation in diamond film. This film is used as an interlayer for subsequent CVD of micro-crystalline diamond (MCD) film. The Rockwell indentation test shows that boron doping could effectively improve the adhesive strength on substrate of as deposited thin diamond films. Dry drilling of graphite is chosen to check the multilayer (BDD + MCD) film performance. For the sake of comparison, machining tests are also carried out under identical conditions using BDD and MCD coated micro drills with no interlayer. The wear mechanism of the tools has been identified and correlated with the criterion used to evaluate the tool life. The results show that the multilayer (BDD + MCD) coated micro drill exhibits the longest tool life. Therefore, thin BDD interlayer is proved to be a new viable alternative and a suitable option for adherent diamond coatings on micro cutting tools.

## 1. Introduction

Graphite is the most widely used material for electrical discharge machining (EDM) electrodes due to its low electrode abrasion, light weight, low cost and especially excellent high temperature endurance in the spark arc when compared with cooper [1,2]. However, tools easily suffer severe wear on their cutting edges due to abrasive properties of graphite powders formed during graphite machining. Consequently, to enhance their cutting performance in the machining of high quality EDM graphite, cutting tools are proposed to be coated on wear-resistant diamond thin films using the hot filament chemical vapor deposition (HFCVD) technique as it is convenient to directly deposit diamond thin films on tools with complex geometries by this method [3,4,5,6]. 

When using CVD diamond films in cutting tool applications, cemented carbides (WC-Co) are commonly used as tool material. The major difficulty in depositing diamond on WC-Co tools arises from the binder Co, which induces graphitization during HFCVD procedure. This would result in poor adhesive strength between substrates and diamond coating and further up restrict the application of diamond coated cutting tools. 

Various approaches have been reported to avoid or reduce this catalyst effect of cobalt on the growth of diamond films. One widely used method is chemical solution etching, including Murakami reagent and acid etching [7,8]. However, some problems remain unsolved as cobalt diffuses from the interior to the surface at high temperature under diamond deposition conditions [9]. Consequently, an interlayer is introduced to deposit between the substrate and diamond film to further enhance diamond coating adhesion and prolong tool life. Different interlayer systems such as Al/TiN/TiCN [10], SiC [11], Cr/CrN [12,13], Co-B [14], and BN [15] have been investigated. In general, interlayers seem to bring qualitative improvement to adhesion enhancements due to its barrier effect against cobalt diffusion. Boron doped diamond (BDD) film may also be an effective barrier to stop cobalt diffusion as cobalt could be fixed in the film due to formation of CoB and Co_2_B during deposition of BDD film [16]. Some research has been conducted to study micro structure, residual stresses, crystalline quality and electrical conductivity of BDD film [17,18]. However, the mechanical properties of the BDD layer [19], especially when it is used as interlayer to improve coating adhesion and cutting performance of diamond coated cutting tools have, to our knowledge, rarely been investigated. Consequently, in this research, we are concerned with the growth of micro-crystalline diamond (MCD) films on WC-Co micro drills that have been pre-coated with BDD interlayer, with an emphasis on the cutting performance enhancement of BDD + MCD coated micro drills compared with MCD or BDD coated micro drills in graphite machining.

## 2. Experimental Section 

### 2.1. Fabrication of Diamond Coated Micro Drills

**Figure 1:**
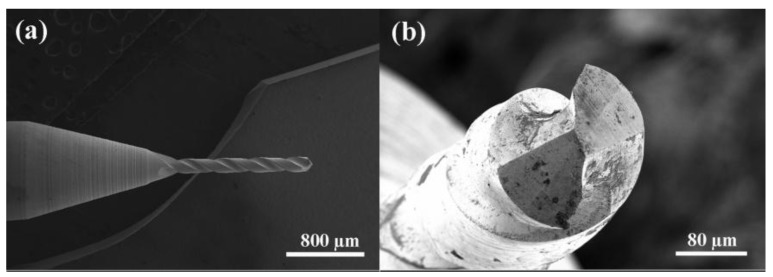
Images of as-received micro drills.

The substrates used in this work are commercial tungsten carbide two-flute micro drills (φ = 0.25 mm) which contain 6 wt % cobalt binder, as shown in Figure 1. The following surface pre-treatments for WC-Co micro drills are used before CVD process: (1) dipping the substrates in Murakami’s reagent (10 g K_3_[Fe(CN)]_6_ + 10 g KOH + 100 mL H_2_O) in an ultrasonic vessel for 5 min; (2) etching substrates with Caro’s acid (30 mL H_2_SO_4_:70 mL H_2_O_2_) for 30 s; (3) ultrasonic treatment in the compound solution of alcohol and diamond powders; (4) ultrasonic washing by distilled water; (5) ultrasonic washing by acetone.

After pretreatment, un-doped and boron doped diamond films are deposited on micro drills using bias voltage enhanced HFCVD apparatuses. Trimethyl borate (B(OCH_3_)_3_) and acetone are used as boron and carbon source, respectively. Trimethyl borate is dissolved in acetone according to a preset boron-substituted carbon (B/C) ratio to obtain boron doped diamond films. The mixed solution is then displaced into a bubbler immersed in glacio-aqueous mix solution to control the flux of carbon and boron source. Ultrahigh purity H_2_ is used as a carrier gas which flows through the mixed solution and is poured into the vacuum reaction chamber. Two triple-twisted tantalum wires with a spacing of 40 mm are used as hot filaments and are set 12 mm above the top of micro drills. The hot filaments are dragged into a straight position by heat-resisting springs.

During deposition, the temperature of hot filaments and cutting parts of micro drills are kept at about 2200 °C and 800–900 °C, respectively. A negative bias is applied to the substrate for enhancing the diamond nucleation density. In our work, three types of diamond thin films, called MCD, BDD and MCD film, with a BDD interlayer are deposited on cutting parts of micro drills. The detailed deposition parameters are listed in Table 1. BDD + MCD composite films are firstly grown in a boron-doped HFCVD reactor and then in a different but otherwise identical HFCVD apparatus that has never used boron-containing gases because boron is known to diffuse into the sidewalls and components within a reactor, and then diffuse out again during later growth runs [17].

**Table 1:** Diamond film deposition parameters.

**Types of diamond film**	**MCD**		**BDD**		**BDD + MCD**		
	Nucleation	Growth	Nucleation	Growth	Nucleation	Growth1	Growth2
B/C atomic ratio [ppm]	0	0	3500	3500	3500	3500	0
H_2_ flow [sccm]	200	200	200	200	200	200	200
Acetone & H_2_ mixed flow [sccm]	80	70	–	–	–	–	70
Acetone & B(OCH_3_)_3_&H_2_ mixed flow [sccm]	–	–	80	70	80	70	–
Pressure [kPa]	1.6	3.3	1.6	3.3	1.6	3.3	3.3
Bias current [A]	0.02	0	0.02	0	0.02	0	0
Duration [h]	0.5	3.5	0.5	3.5	0.5	1.5	2

### 2.2. Characterization

After deposition, the surface morphologies of these films on micro drills are captured by field emission scan electron microscopy (FESEM, Carl Zeiss, Germany, Ultra 55). The ingredients and purity of MCD, BDD, BDD interlayer and BDD + MCD films are examined by Raman spectroscopy (SPEC14-03). Rockwell indentation tests are conducted on WC-Co samples to evaluate the adhesion of these coatings by a Hoytom indentation instrument. The conical Rockwell intender is forced into the coating with a load of about 1500 N.

### 2.3. Drilling Tests

Cutting experiments are conducted for as-fabricated diamond coated micro drills on a CNC carving machine. The workpiece is cuboid shaped high quality EDM graphite of grade ISO-68 (Toyo Tanso Inc., Troutdale, OR, USA) with density of 1.88 g/cm^3^. Suction tubes are applied during machining to sweep away graphite powders. The spindle speed, feed rate and depth of drilled holes are 15,000 r/min, 200 mm/min and 1.3 mm, respectively. After drilling a certain amount of holes, main cutting edge of micro drills are photographed by FE-SEM equipped with measuring system. For each tool, 10 locations of wear land on its flank face are measured and the averaged value is regarded as its flank wear (VB).

## 3. Results and Discussion

### 3.1. Characterization of Diamond Coated Micro Drills

**Figure 2:**
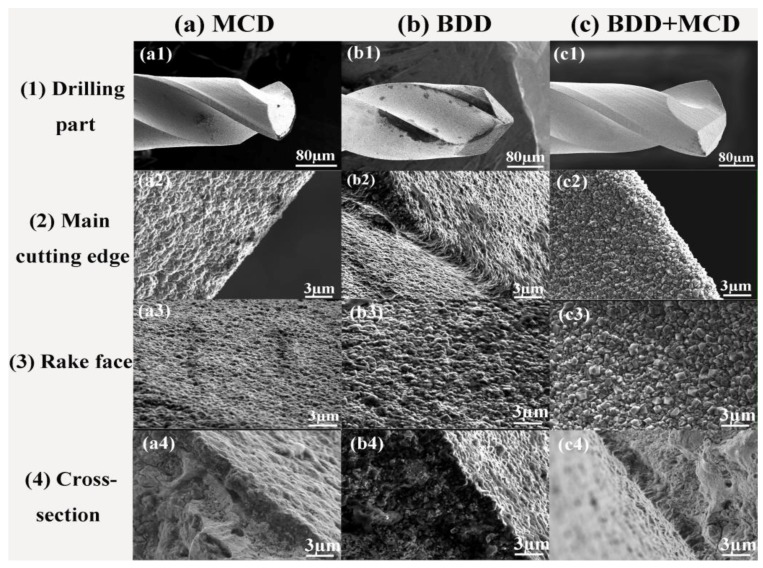
Field emission scan electron microscopy (FE-SEM) images of as-fabricated (**a1**–**a4**) micro-crystalline diamond (MCD); (**b1**–**b4**) boron doped diamond (BDD); (**c1**–**c4**) BDD + MCD coated micro drills.

The surface morphologies of as-fabricated diamond coated micro drills are investigated using FE-SEM. Figure 2 shows the FE-SEM images of drilling part (a1,b1,c1), main cutting edge (a2,b2,c2), rake face (a3,b3,c3) and cross-section (a4,b4,c4) views of diamond coated micro drills with a different amplification factor. It could be observed that a continuous layer of diamond crystallites with average grain size of 1–3 μm is uniformly coated on different locations of these micro drills. Comparing the morphologies of diamond films, it can be seen that most diamond crystallites of BDD + MCD composite film have a hexahedral shape while that of MCD and BDD film have a sharp octahedral shape. From the cross-section views of these micro drills, it can be observed that the thickness of diamond layer is all about 2 μm after 4 h deposition time. This means after film deposition, the cutting edge radius of these micro drills are still almost the same. The difference of their cutting performance is only attributed to the properties of the as-deposited films.

**Figure 3:**
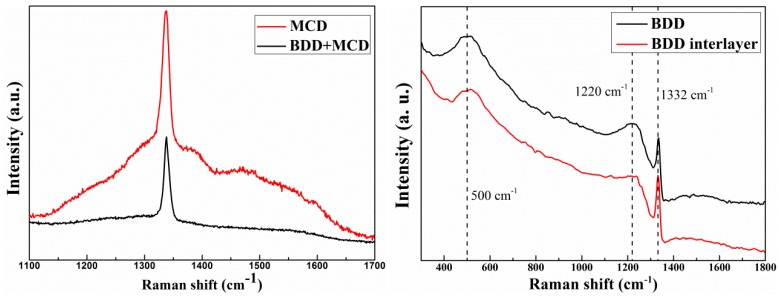
Raman spectra of as-deposited diamond films on micro drills.

The quality of as-deposited diamond coatings is examined by Raman spectroscopy, using a He–Ne laser with an excitation wavelength of 632.8 nm. Raman spectra of these thin films are plotted in Figure 3. The spectrum of MCD film features a sharp peak at 1332 cm^−1^, characteristic of high purity sp^3^ C–C bonding. The broad peak at 1400–1600 cm^−1^ represents that there are non-diamond phases, including graphite or amorphous carbon, in the film. However, no other peak is visible besides the 1332 cm^−1^ diamond peak on Raman spectrum of BDD + MCD composite film, which indicates the high purity sp^3^ C–C bonding in the outer MCD layer. The different amount of graphite or amorphous carbon in these two MCD layer may be that during diamond film deposition, boron dopant could effectively stop binder Co diffusing from tool interior to the surface at high temperature, thus suppress the graphitization of MCD layer on BDD interlayer [9,20].

In the case of BDD film and BDD interlayer, the as deposited diamond films are examined by Raman spectroscopy in the region of 300–1800 cm^−1^ in order to get more information of the boron doped diamond films. It can be seen that the symmetric Lorentzain peak at ~1332 cm^−1^ characteristic of undoped diamond changes to an asymmetric Fano-like lineshape because of a quantum mechanical interference between the zone-center Raman-active optical mode and the continuum of electronic states induced by the high concentration of dopant [17]. Another feature related to the boron incorporation is the shift of the diamond peak to lower wavenumber (1331.09 cm^−1^ for BDD films and BDD interlayer), accompanied by two broad bands around 500 cm^−1^ and 1220 cm^−1^ [21,22,23,24]. These bands may be associated with the actual boron incorporation in the diamond lattice [25] and the band at ~500 cm^−1^ is related to the vibrational modes of boron pairs due to the distortion in the diamond lattice caused by these isolated defects [26].

### 3.2. Results of Rockwell Indentation Test

**Figure 4:**
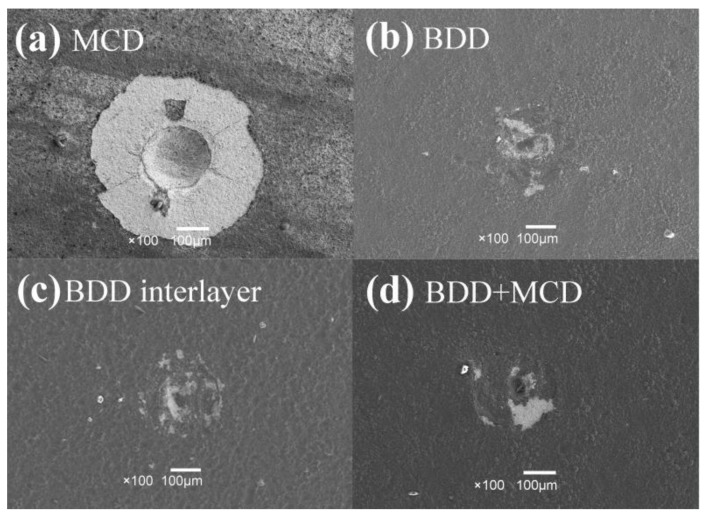
Coating failure modes of MCD (**a**); BDD (**b**); BDD interlayer (**c**); and BDD + MCD (**d**) coatings after indentation tests.

The Rockwell indentation tests are conducted on the MCD, BDD, BDD interlayer and BDD + MCD coated tungsten carbide samples to evaluate their respective interfacial coating–substrate adhesion strength. It can be seen in Figure 4 that detached coating around the indentation is visible on MCD (Figure 4a) film after indentation. There are only micro cracks at some areas and no coating delamination in the case of BDD (Figure 4b), BDD interlayer (Figure 4c) and BDD + MCD (Figure 4d) films. It suggests that the adhesive strength between BDD, BDD interlayer and BDD + MCD composite films and substrates is much higher than that of MCD film between substrate. This could be ascribed to interaction between Co and boron to form CoB and Co_2_B in BDD layer. Meanwhile, these compounds could effectively stop cobalt from moving outside the substrate during the deposition procedure.

### 3.3. Results of Drilling Tests 

**Figure 5:**
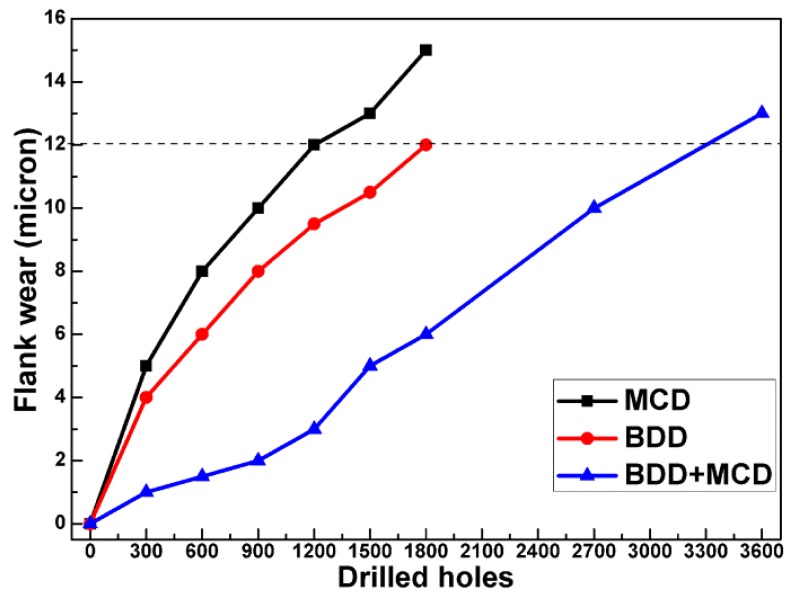
Flank wear for MCD coated, BDD coated and BDD + MCD coated micro drills as a function of drilled holes.

The effectiveness of BDD interlayer on cutting performance of diamond coated micro drills is investigated in dry drilling of high quality EDM graphite. For the sake of comparison, pure MCD and BDD coated micro drills are involved. Figure 5 shows the flank wear for all examined micro drills as a function of number of drilled holes. 

In graphite machining, brittle fracture of polycrystalline aggregates takes place instead of plastic deformation in metal machining. Thus, the tool failure mode in graphite machining is mainly abrasive wear of graphite powders. As abrasive wear has the largest influence on flank wear [27], the main wear type of cutting tools involving graphite machining is flank wear, which is mainly induced by abrasive wear between graphite particles and the flank face of the tools.

As exhibited in Figure 5, the flank wear of these coated micro drills increases steadily with the number of drilled holes. Note that the rising rate of flank wear on MCD or BDD coated micro drills is somewhat higher than that of BDD + MCD coated micro drills. After drilling 1800 holes, the flank wear of MCD and BDD coated micro drills rises to 15 and 12 μm. Comparatively, BDD + MCD coated micro drills show flank wear of only 6 μm. Subsequently, only BDD + MCD coated micro drill is used to drilling graphite and its flank wear is comparable with that of BDD or MCD coated micro drills until 3600 holes are drilled. If flank wear of 12 μm is chosen as the criterion of tool life, it could be concluded that tool life of BDD + MCD coated micro drills is 2–3 times longer than that of BDD and MCD coated micro drills.

**Figure 6:**
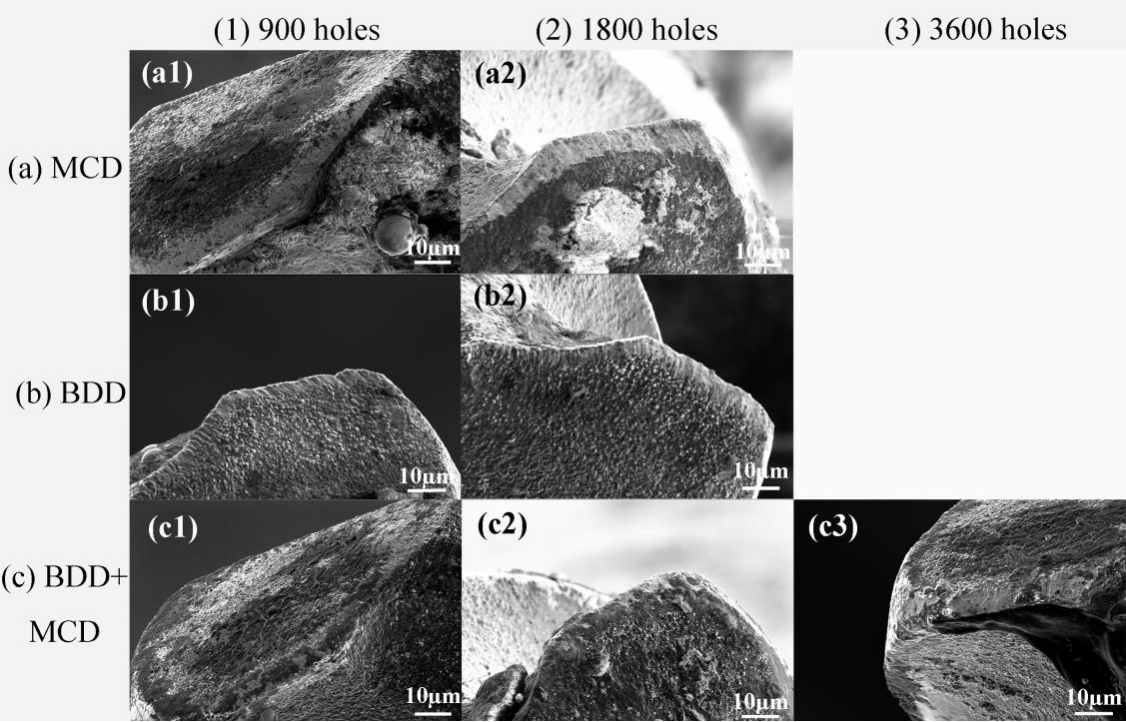
Images of main cutting edges for (**a1**,**a2**) MCD; (**b1**,**b2**) BDD; and (**c1**–**c3**) BDD + MCD coated micro drills after drilling (**a1**,**b1**,**c1**) 900; (**a2**,**b2**,**c2**) 1800; and (**c3**) 3600 holes.

To assess the wear mechanism of these coated micro drills, FE-SEM images of main cutting edges are captured after drilling a certain amount of holes, as shown in Figure 6. After drilling 1800 holes (Figure 6a2,b2,c2), it can be seen that flank wear of MCD (Figure 6a2) coated micro drill is comparable with that of the BDD (Figure 6b2) coated micro drill, but their wear types are quite different. In wear land of MCD coated micro drills, a large area of diamond film is peeled off from the substrate, which may be attributed to the relatively poor adhesive strength between MCD coating and substrate. However, no obvious coating delamination could be seen on the flank face of the BDD coated micro drill. The wear land on this micro drill is mainly composed of chipping on cutting edge and abrasion grooves polished by graphite powders. It suggests that doping boron in diamond coating could effectively improve adhesive strength between diamond coating and substrate. This result is in accordance with findings in Raman spectra and indentation test. 

It is noted that much less flank wear could be seen on BDD + MCD (Figure 6c2) coated micro drill compared with MCD (Figure 6a2) or BDD (Figure 6b2) coated micro drills after drilling 1800 holes. On the one hand, the cutting performance enhancement of BDD + MCD coated micro drills compared with MCD coated ones can be ascribed to that BDD interlayer improve the adhesion between diamond coating and substrate. On the other hand, the cutting performance of BDD + MCD coated micro drills is better than that of BDD coated ones. This can be assumed to be due to the maintenance of wear resistance of diamond film. As the boron incorporation between diamond grains in CVD diamond layer would reduce the purity and quality of diamond thin films to some extent, resulting in lower hardness and Young’s modulus of boron doped diamond (BDD) film compared with un-doped diamond film [28,29]. In general, materials with high hardness and stiffness have high wear resistance. Consequently, the wear rate of BDD film is higher than that of un-doped MCD film. Meanwhile, there is barely a mismatch of coefficient of thermal expansion between BDD interlayer and MCD coating, which ensures good adhesion between BDD interlayer and MCD film. That is to say, BDD + MCD coated micro drills combine the good incorporation between substrates of BDD interlayer and good wear resistance of un-doped MCD layer. Therefore, BDD interlayer before deposition of MCD film on micro drills is efficient in further improving cutting performance of diamond coated micro drills and extending the application of diamond coating into the micro scale. The result is useful for broadening the application area of BDD film in the mechanical processing field.

Sequentially, only BDD + MCD coated micro drill is used to drill graphite, its flank wear begins to rise up and diamond films are found peeling off at some locations after drilling 3600 holes, as can be seen in Figure 6c3. At this time, the flank wear of BDD + MCD coated micro drill is comparable to that of MCD or BDD coated micro drills. That is to say, this novel BDD + MCD composite film could effectively enhance cutting performance of these micro drills compared with MCD or BDD monolayer film.

## 4. Conclusions 

MCD, BDD and BDD + MCD composite films with thickness of about 2 μm are uniformly coated on cutting part of the micro drills. The broad peaks around 1220 cm^−1^ and 500 cm^−^^1^, which are the feature of boron incorporation in the diamond lattice, are visible in Raman spectrum of BDD coating and BDD interlayer on micro drills. Results from Raman spectra show that the purity of MCD on the BDD interlayer is higher than that of MCD without the BDD interlayer. Detached coatings around the indentation can be seen on MCD film, while only micro cracks are observable on BDD coating and BDD interlayer after indentation test. That is to say, boron doping could effectively improve adhesion between diamond film and substrate. In dry machining of graphite, the tool life of BDD + MCD coated micro drill is about 2–3 times than that of MCD or BDD coated micro drill. BDD interlayer before MCD deposition could effectively enhance cutting performance of diamond coated micro drills. The main wear type of BDD + MCD coated micro drill is flank wear. However, chipping and coating delamination could also be observed in addition to flank wear on BDD and MCD coated micro drills. The difference of these wear types is attributed to relatively good adhesive strength between substrate but low wear resistance of BDD layer and relatively poor adhesive strength between substrate but high wear resistance of MCD layer. 
